# The Pathology of Fever

**Published:** 1899-08

**Authors:** J. Clarence Johnson

**Affiliations:** Atlanta, Ga.; Professor of Physiology and Pathological Anatomy Atlanta College of Physicians and Surgeons


ATLANTA

### Journal-Record of Medicine.

Successor to Atlanta Medical and Surgical Journal, Established 1855,
and Southern Medical Record, Established 1870.

VOL. I. AUGUST, 1899. No. 5.

BERNARD WOLFF, M.D., DUNBAR ROY, A.B., M.D., EDITORS.

M. B. HUTCHINS, M.D., BUSINESS MANAGER.

PUBLISHED MONTHLY. OFFICE 305 AND 306 FITTEN BUILDING.

### ORIGINAL COMMUNICATIONS.

### THE PATHOLOGY OF FEVER.

BY J. CLARENCE JOHNSON, M.D.,

Professor of Physiology and Pathological Anatomy Atlanta College of Physicians and Surgeons.

[NOTE.—In 1895 I read before the Atlanta Society of Medicine, a paper on "The Pathology of Fever" in which some features of the present article were discussed. Since that time I have pursued the study of the subject, and have, I think, reached a correct definition of fever. It was not until December, 1898, that I completed the present paper. The chief facts which it embodies were delivered in lectures to the graduating class of the Atlanta College of Physicians and Surgeons during the session of 1898–99, and were subsequently discussed before the Atlanta Society of Medicine.

I was gratified to learn, in a recent interview with Dr. William Henry Porter, of New York, that he entertained views similar to those which I had advanced, and had published, in April, 1899, an article entitled "Febrile Conditions: Their Relation to Body-Heat Production and Their Treatment." In this article Dr. Porter demonstrates that fever is not the result of increased combustion, but is attended by diminished heat-production. He assumes also that it is due to deficient elimination of heat. I have not agreed entirely with the last idea, but have undertaken to explain the abnormal rise of temperature by the disturbance of the relation of chemical and physical forces to physiological action.]

In entering upon the composition of this paper, I am not unmindful of the magnitude of my undertaking and the utter futility of attempting to comprehend, even within the liberal space allowed, the extensive range of facts pertinent and indispensable to a clear and exhaustive treatise of the subject.

The intimate relation, both causative and sequential, which fever bears to many forms of disease, presupposes an incomplete and imperfect study of its pathology, without that distinct nosological classification which the diseases themselves possess. The task would be easier were I to select for discussion a particular type of fever, but such limitation would hinder or defeat the accomplishment of my purpose and lessen the value of the result.

It would doubtless enhance the interest of the paper to note, in passing, and apply to points in question various views relevant to the subject which have been advanced from time to time by observers of eminent ability and scientific attainments; but, with the deference due these worthy teachings, I desire to present solely such ideas as have been suggested by clinical experience and elaborated and developed by application and comparison of established physiological and pathological facts.

One of the first questions asked by a patient of his physician is: "Have I any fever?" Next, "Is it very high?"

It is true, I believe, that the presence or absence of fever, and the degree, in a majority of acute diseases and in some chronic as well, is regarded as a valuable index not only to the peculiar nature of the disease, but to the condition of the patient and the probable course, duration and severity of the attack. Baffled by symptoms that are vague or obscure, we are often prone to satisfy ourselves and console the patient with the fact that he has no fever, or that it is very slight. Mr. A. is better to-day; his fever is not so high. Mr. C. is not so well; his fever is a little higher.

What is the cause and meaning of this faith? Is fever a relentless foe, in league with death, or a perverted phase of nature? Does it hold the key to man's vital storehouse, or stand sentry at life's portals to repel or invite dissolution or decay?

I will not stop, in anticipation, to debate the theories that it is a conservative process designed to combat the operations of its cause, but pass to the proper study of the subject itself, leaving their refutation or confirmation to facts which I will attempt to establish.

Fever is such a common attendant of disease that we are never surprised to find it, and too seldom stop to inquire why it is present. It is considered important or not important according to the supposed cause, probable course and degree. It always denotes a departure from the healthy state.

Fever is a continued elevation of temperature above the normal. The thermometer in the mouth registers 99½° F. We say that our patient has one degree of fever. We may not know the cause, and there may be no apparent lesion; still we pronounce him ill.

We examine a plaintiff in a suit for injury, or any one whose interest creates a desire to be considered ill. He complains of various aches and pains, but we detect no deviation from the normal state of health, and so testify in court. A patient applies for relief from vague discomfort, malaise, disinclination to accustomed efforts. We succuss, percuss, and auscultate, but detect no abnormality. We place the thermometer under his tongue, and it registers 100½° F. We pronounce him ill, and write a recipe for him. A child is bright and playful in the morning; in the afternoon, without discernible reason, is flushed with fever; the next day is as well as ever, and there is no appearance or suggestion of the cause. What is the nature of its illness? It has fever, but what is fever? That is the question.

Pathology is the physiology of perturbated action, and we cannot understand the phenomena of disease without a knowledge of the conduct of the natural functions or function whose perversion led to its development.

The process of nutrition, secretion and excretion involves various physical and chemical conditions and forces, which operate alike within and without the body. These forces and conditions are likewise subject to internal and external influences which vary their physiological as well as mechanical action and state.

Let us look, then, for a moment at some of the laws governing these forces and conditions. When we speak of heat of the body we usually mean the amount. Temperature refers to the degree of heat. A rod of iron one foot long and one inch in diameter will contain more heat of the same degree than a rod of iron one-half foot long of the same diameter and same degree. So a cup of water may have the same temperature as a bucket of water, but the bucket of water will contain more heat than the cup of water.

Heat is convertible into kinetic energy, and, *vice versa*, energy may be exhausted in the form or production of heat. The dynamic relation of heat and energy is measured by their effect upon matter. It has been demonstrated that the heat required to raise one pound of water 1° F. is equivalent to 772 feet pounds, or sufficient to raise 772 pounds one foot. Hence the amount of heat a body contains determines the amount of its potential energy.

The bar of iron, for instance, one-half foot long, will contain one-half the potential energy that the rod one foot long will contain. So, to equalize their potential energy, the temperature of the one must be elevated twice its degree, or the temperature of the other lowered one-half.

The *vis viva*, or energy, then, of the body is commensurate with the amount of heat which, converted into force, is or can be utilized by its molecules to maintain their relative position in the mass, or in change of state, and resist the opposition of external influences or effect external conditions.

A reduction of the temperature of a body, then, is equivalent to a reduction of its energy, and an elevation of the temperature is equivalent to an increase of its energy. The application of this energy or force by its molecules as kinetic or potential is determined by the character of their relation in the mass which they constitute and the direction in which it is employed, which, in turn, is governed by the nature of the opposition to be overcome.

Water boils at 212° F., under certain conditions of pressure and altitude. When it reaches this point—its caloric capacity—it is dissipated as steam or vapor. This steam or vapor is applicable as force or propelling power, as in the movements of a steam engine.

Before this potential energy, however, can be so employed, a part must overcome the opposition of the molecules of water themselves, and the tubes surrounding the mass. Hence, the effect of steam will be measured by the nature and size of the vessel in which it is generated and the amount of water contained.

Applying these facts to the subject under discussion, we will be better able to understand the relation of thermogenesis to the animal and organic functions.

The temperature of a child and a man is the same in health—though the body of the man contains more heat than that of the child, and, hence, more potential energy. But the work to be accomplished in the body of the child, while of the same nature as that in the body of the man, meets with less, though proportionately more opposition, and hence less is required.

The temperature of the body in summer is the same as in winter, but the performance of the vital functions, and maintenance of vital capacity meets with more resistance in winter than in summen, hence more heat is generated and more energy is expended. The temperature of the body is the same in the torrid as in the temperate zone, yet the thermal conditions of the atmosphere differ widely in these localities.

The temperature of the body is the same at sea level and on the mountain, though the atmospheric pressure, humidity and temperature of these localities vary to a great degree, and, to the extent of this variation, influence the general functions of the animal organism.

The temperature of the body, save for the slightest variation, is the same in the morning as in the evening; in the active, as in the quiescent state.

Why are these facts true? Because the body is composed of proximate principles which are originally derived from the inorganic world, the form and function of these proximate principles being dependent upon the operation of the same forces, physical and chemical, which govern their union and action without the body, both in their simple and compound relations. Hence, the various organs and tissues which they constitute are vested with the essential properties they embody, and are susceptible to the same influences and conditions.

As all matter has a caloric habit and capacity, so the human body has a habit and capacity of heat absorption, production and liberation, subject to modifications, which vary with the physical and chemical changes in the elements employed in the performance of its functions and in the process of repair. When such change is abnormal, its result is elevation or decline of the temperature. If elevated, it is called fever.

We lay down, then, as a premise, that whatever causes fever, does so either by abnormal increase in heat production, deficient heat elimination or consumption, or by perversion of them all. The amount of heat generated may be consistent with the thermogenic function of the body, yet deficient appropriation of it as energy may cause an increase beyond the natural power of elimination. Likewise, less heat may be generated than is consistent with the caloric capacity and requirements of the body, and the degree will remain the same, in the event of deficient elimination. Thus it has been observed that when the body is subjected to a high external temperature, its temperature remains normal so long as there is preserved an even balance of its own product and that supplied from without consistent with its needs. The same fact applies where the body is subjected to a low temperature. More heat is required, more is generated, less is set free.

The amount of heat in the human body is estimated by the amount of latent heat which the molecular masses of proximate principles contain, or surrender or absorb in change of state, the amount concealed or in use as kinetic energy in the metabolism of the cells and osmotic interchange between the solids and fluids, and that additional required for the maintenance of the physical condition essential to the performance of the general functions.

Reviewing, briefly, the phenomena of cell genesis and reproduction, and bearing in mind the fact that each cell thus formed is endowed with the peculiar powers that gave it birth, growth and development, and constitute it a complete and independent physiological unit, we can more easily explain the *modus operandi* of the mutual perversion and reaction of cells upon each other. The question then resolves itself into a determination of the natural sources of heat in the body, and manner of operation of a cause that interferes with its production, conversion into energy, or its elimination.

Various theories have been advanced explaining the source of heat in the body, but it is now a generally accepted fact that it is produced by combustion of the tissues of the body in the physiological process of animal and organic functions—the union of oxygen with the carbohydrates, hydrocarbons and proteids. The extent of this combination varies with the amount of oxygen taken into the blood, and the extent of its union with these proximate principles. In normal nutrition waste and repair are equal, and, while the amount of kinetic and potential energy created by the oxidation in various organs may be more or less at given periods, the aggregate of the *vis viva* remains the same by the exercise of compensatory functions.

In a state of health, therefore, the temperature is not influenced by increased generation of heat—the excess being partly employed in the chemical and physical action of the organisms, and that not so used is set free. For example, it has been demonstrated that prolonged and active muscular exercise causes marked increase in the production of heat, and physiologists have undertaken to prove that the thermic changes are due to the metabolism or oxidation of the muscle protoplasm. But, while the elimination of $CO_2$ and water is increased, the amount of urea is not consistent, nor is the temperature elevated but slightly and for a brief period.

Hence we are forced to the conclusion, that, while the chemical and physical changes incident to muscular contraction does cause an increased heat production, the part contributed by the protoplasm of the muscle is its pro rata in the general physiological process, and that the heat it delivers is not derived from its kinetic alone, but, also, from its potential energy. As further confirmation of this fact and its bearing upon the pathology of fever, it is worthy of remark that in the febrile state the voluntary system of muscles is at rest, and the potential energy which it exercises or expends is only that employed in the metabolic and osmotic processes of nutrition. Additional proof of this fact will be advanced in the course of the discussion, and special application made, but it suffices now to simply give it mention as one of the factors of normal heat production, and the part which it might play in the development of any pathological condition.

One of the most fertile sources of heat in the body is the glandular system. Stimulation of the salivary glands is followed by an increased metabolic or catalytic action of these organs, and while this metabolism varies according to the manner and source of stimulus, and its channel of transmission, and there is also a difference in the relative proportion of the ingredients of the secretion, the product presents the essential characteristics of saliva and is attended by an elevation of temperature in the part.

The factors entering into the formation of these products, physiologically speaking, are the same as in muscular contraction, in active intellection, or any other function or process in the body.

We have already seen that before any matter is capable of exercising potential energy it must possess a requisite amount of latent heat or force. Were this not true the metamorphosis would be destructive instead of constructive, and the effect upon the cell would be atrophic instead of reparative.

It is obvious, then, that the amount of heat generated and contained by the body will be determined by the amount of latent caloric and specific capacity, and the full caloric potential—the latter being dependent upon the sum and relation of the former two—and that whatever reduces or increases the potential energy or kinetic force, and hence the amount and degree of heat, must do so by interference with the metabolism of the cells. This is equally true of its production, consumption or dissipation, because the avenues of elimination by which, under normal conditions, the temperature is regulated, are parts of the process just described—all receiving their power from the electro-vital conversion of heat.

Irritation of an end filament of a nerve will cause a determination of blood to the part which it supplies, and the hyperemia thus induced will cause increased tissue metamorphosis with increased production of heat, but the total of the product of the metamorphosis is equal in material formation, urea, $CO_2$, $H_2O$ and protoplasmic substance, secretion, energy and heat, as the pabulum extracted from the blood, the oxygen consumed and the kinetic or chemico-vital force expended in the metabolic change. The temperature of the part is slightly elevated during the metamorphosis, but returns to the normal or more general degree *pari passu* with the subsidence of the process. This return is due to a discharge through the circulation of the organ—by blood-vessels and lymphatics—of the excess of heat not employed in the physiological action nor reserved as potential or vital energy by the cells as compensation for that consumed in the performance of their increased function and nutrition, which caused the elevation.

The temperature in the right heart is 38.8 C., while it is only 36.8 C. in the superior vena cava, which is in intimate anatomical relation with the right auricle and ventricle. So, a simple increase in the production of heat or transient elevation of the temperature cannot be classed as pathological. But should any organ or part of the body, from any cause or in any manner, produce more heat than is essential to its own catalytic and metabolic action, involving its physiological function and nutrition, and the product of the oxidation or metamorphosis is not commensurate with the form material used, the oxygen consumed and energy expended, such action is abnormal and disturbs the relation which that organ or part sustains to other organs of the body.

For example, the liver is an organ of excretion and secretion, in a constant state of activity, and hence is one of the chief sources of heat in the body. The temperature in the hepatic vein is usually 107° F. This increased amount of heat is due to its nutritive, biliary and glycogenic functions. In the performance of these functions a large amount of potential energy is consumed, and this energy is taken from the aggregate of the vital forces of the body. For the preservation of the balance of the economy it is necessary that the liver should return to the body at large the equivalent in pabulum, laden with latent force and actual heat of that which it consumes. Hence there is delivered to the right side of the heart, through the hepatic veins, various proximate principles ready for future transformation, and an excess of heat which elevates the rtempeature of the blood passing to the heart and through the lungs, to equalize the temperature of that which is to be distributed through the systemic circulation.

Anything which increases the functional activity of the liver, as irritation of the fourth ventricle, or stimulation of the vagus, or the hepatic plexus, will cause an increased oxidation of carbohydrates and proteids, and consequently an increased excretion of bile, formation of glycogen, urea, $CO_2$ and generation of heat. Should there be a deficient elimination of heat by the lungs from any cause, the excess which they receive will be distributed throughout the body and, eventually, escape through the various emunctories. Thus is the normal amount of body-heat and temperature maintained. Should the hyperemia continue and inflammation ensue, the physical condition which attends this morbid process opposes the chemico-vital action and osmotic interchange of the blood-vessels and hepatic cells, and hence their nutrition and functions are impaired, their kinetic force becomes practically potential, and, free from proper exercises, loses its normal relation to all metabolic and catalytic action.

As a consequence of this perversion effete material, which is usually extracted from the blood by the liver, remains in the general circulation, and pabulum, which needs, and is wont to have, further transformation to fit it for the reconstructive functions and support of the body, is checked in its course and adds to the stagnant mass, or retreats through the portal system to hinder the various processes of digestion and absorption in the alimentary tract.

If we will pause for a moment to consider the histology of the liver in this inflamed condition, we will readily perceive that oxidation is really diminished, rather than increased, and that less heat, physiologically speaking, is generated by it than usual.

The physiological action of any organ or tissue of the body depends upon a proper amount of blood of normal quality passing to and through its capillaries within a given period. The amount of blood passing to and through a part depends upon the total amount, state and character of the blood in the body, the condition of the vascular system, especially the vessels supplying the part,—the nervous influence, and the action of the constituent cells. One of these factors cannot act independently of the other, nor can one be perverted without disturbance of the other.

The physiological action of an organ consists in the maintenance of its nutrition and the performance of its function. These processes involve an osmotic interchange between the protoplasms of the cells and the adjacent blood. This osmotic interchange is accomplished by the chemical and molecular affinity of elements that constitute the fluids and solids of the part, and varies as these elements and molecules vary in their composition and relation. Their relation and action are, alike, in a measure, dependent upon and governed by the amount and degree of heat which they contain. The total of the influence and agencies employed in the osmosis is equivalent in effect to the kinetic energy expended.

Hence, a normal osmotic action requires a normal pressure of blood, a normal vessel wall, and normal state of the surrounding cells.

In inflammation, there is stasis; the arterioles and capillaries are distended to twice or three times their natural size; the texture of their walls is changed; their porosity is increased; the liquid constituents of the blood, the leucocytes and some red disks pass through into the surrounding cellular tissue, and intrude upon the habitat of the cells; less oxygen is consumed; less constructive metamorphosis occurs, save in parts; less heat is generated, and the temperature may fall below that of tissues which are adjacent.

But the general temperature is increased. Why is this? That it is caused by the abnormal condition just described is easily apparent. Yet the more important question, how it causes it, remains to be answered.

Incident to the inflammatory process, morbid impressions are received by the end filaments of sensory nerves participating in the perverted action, and by them are exaggerated and transmitted to their centers, whence they are reflected throughout the body, each organ and tissue contributing its quota of vital resistance to their tolerance. To the extent of this contribution is the general trophic and functional action impaired. As evidence of this, let us look for a moment at the febrile state which ensues. All secretions and excretions are diminished in amount; less food is taken into the stomach; digestion is imperfect; absorption is restrained, and nutrition suffers in proportion to the lack of pabulum thus withheld. Compensatory functions are all engaged to supply the needs of organic life; voluntary action is economized; every power is enlisted to combat the insidious decay, yet waste exceeds repair, the temperature rises higher, and remains until nature is subdued, or reasserts her sway.

Yet, however clear the morbid anatomy of inflammation, and however intimate its causative relation to the febrile state may appear, we are aided but little in determining why the general temperature rises and remains above the normal.

It is not difficult to understand how a perversion of one or all of the factors in heat production may lead to this result, but the pathology of fever involves more than simple thermogenesis. It involves not only the independent and relative action of the blood, nervous system and the cells, but the exact part which each plays in the morbid process, and the physical and chemical phenomena which accompany or result from this process.

*(To be continued.)*

